# Factors affecting unmet healthcare needs in female baby boomers: Andersen model application in Korea

**DOI:** 10.1371/journal.pone.0286425

**Published:** 2023-06-01

**Authors:** Min-Jeong Park, Mi-Young Chung

**Affiliations:** 1 Department of Nursing, Kunsan National University, Kunsan-si, Korea; 2 Department of Nursing Science, Sunmoon University, Asan-si, Korea; NUST: National University of Sciences and Technology, PAKISTAN

## Abstract

This study aimed to measure unmet healthcare needs and investigate the factors affecting them in female baby boomers (individuals born between 1955 and 1963) using the Korea Health Panel Data 2017 from February to June 2017 by the Korea Institute for Health and Social Affairs and the National Health Insurance Corporation. The data were analyzed using descriptive statistics, chi-square test, t-test, and multiple logistic regression using SPSS WIN 25.0 program. The results showed that the proportion of unmet healthcare needs was 11.1%, and the primary reason for unmet healthcare needs was the lack of visitation time. Female baby boomers experienced more unmet healthcare needs when they had no spouse (1.63 times), eating problems (2.33 times), and stress (1.31 times). This study is significant because it measured the unmet healthcare needs of women in the baby boomer generation and identified the factors influencing unmet healthcare needs. The study’s results can help provide essential data to decrease the unmet healthcare needs of female baby boomers.

## Introduction

### Study necessity

Unmet healthcare needs are defined as cases wherein healthcare service needs are not met for any reason [1, 2]. In Korea, all populations have been covered by the National Health Insurance Service (NHIS) since 1989, making it easier to use healthcare services. Nevertheless, the rate of unmet healthcare needs is 8.8% [3]. Compared to the mean number of 28 countries in the European Union, it is approximately 3.5-fold higher [4, 5]. It is not easy to check whether a country’s health care system meets the health needs of its members; however, unmet medical care has been used as an alternative for this purpose [6]. The measurement of unmet healthcare needs was very subjective, according to Song et al., because an individual had measured their subjective perceptions of those needs [7]. However, assessing the subjectivity of unmet healthcare needs from the users’ perspective is crucial [8]. Andersen’s model of the health care use behavior is one of the most popular models for studying underuse, misuse, or abuse of the health care system [9]. According to Andersen, individual health care use behavior varies depending on sociodemographic factors, accessibility factors for using healthcare services, and direct factors of health care use [10].

Studies applying the Anderson model have shown that factors affecting unmet healthcare needs were age, sex, marital status, education level, income level, economic activity, type of health insurance coverage, chronic illness, disability, depression, and subjective health conditions [11, 12]. Women have been reported to have higher unmet healthcare needs than men. Additionally, studies have reported that individuals who were older, had lower levels of education, were employed, had lower income levels, had poor health conditions, had chronic diseases, depression, stress, and limitations to daily routine had higher unmet healthcare needs [7, 11]. Individuals experiencing unmet healthcare needs may conclude that the healthcare system is ineffective and may negatively perceive the public healthcare system. Additionally, they miss the most appropriate time to receive medical treatment, resulting in a higher severity of disease and increased prevalence rate of chronic disease, which may lead to increased healthcare costs [1, 13]. In response, the Korean government offered transportation for low-income older people and other vulnerable groups who have difficulty moving around due to disabilities. It also promoted medical equity by addressing issues that contribute to unmet medical needs, such as providing home visit services to manage the health of people with chronic diseases [14, 15]. Additionally, the unmet medical needs of different classes are identified through regular medical panel surveys carried out by national institutions, and solutions to the medical use inequality are being sought.

Meanwhile, as baby boomers enter their senior years, the structural change in Korean older adults has accelerated. They are the post-Korean War generation born between 1955 and 1963, with large population groups representing 14.6% of the total population in South Korea. They are a core generation who contributed to Korea’s rapid economic development, experienced foreign exchange and financial crises, and generously sacrificed for parents and children [16]. After the baby boomers entered the 65-and-older age group since 2020, older adults aged ≥ 65 years accounted for 15% of the total population. As of 2021, they accounted for 16.5% of the overall population, and Korea has rapidly entered an aging society [17]. Entering the old age population will diversify this age group and may have a significant ripple effect throughout society, such as in the labor market, social services for old age, and healthcare services. Moreover, several problems may surface because of old age; therefore, a rational policy should be established [18]. With increasing life expectancy, baby boomers are expected to live 30 more years. However, they retire without preparing for their old age because of the burden of raising children and supporting their parents [19]. This threatens physical and mental health by causing a loss of social position and role and decreasing self-esteem [18].

The female baby boomer generation has a relatively higher education and income level than the current older generation. They change their attitude toward old age and consider entering old age as a new beginning of life and a chance to accomplish their self-realization [20]. Furthermore, during the transition from middle age to early old age, they attempt to manage their physical and mental health actively [18]. Easy access to and use of required healthcare services are essential for good quality of life and the resolution of health problems [21]. Specifically, since healthcare services are the main policy that can resolve health inequalities in older adults, they should be thoroughly modified [12, 22].

Therefore, it is essential to establish health policies that reflect women’s diversity in the early phases of old age by confirming female baby boomers’ perspectives on healthcare needs. Accordingly, the current study aimed to investigate the factors affecting unmet healthcare needs and contribute to developing policies for decreasing unmet healthcare needs in female baby boomers.

### Study purpose

This study aimed to analyze factors affecting unmet healthcare needs in female baby boomers using data from the Korea Health Panel Survey 2017, and detailed objectives are as below:

1. To analyze unmet healthcare needs in female baby boomers and the causes.
2. To identify characteristics of unmet healthcare needs in female baby boomers.
3. To investigate factors affecting unmet healthcare needs in female baby boomers.

## Methods

### Study design

Secondary data analysis was conducted to investigate the current status of unmet healthcare needs in female baby boomers in Korea and identify factors affecting these using the Korea Health Panel Survey 2017.

### Study participants and data collection

This study used data from the Korea Health Panel Survey 2017 (Version 1.6), a nationwide investigation jointly conducted by the Korean Institute for Health and Social Affairs and the NHIS annually since 2008. Data from the Korea Health Panel Survey are used as primary data for establishing and implementing government policies, such as the current status of public health, medical expenditure, health level, and health behavior.

Following the regulations of the Korea Health Panel Survey, we received data via e-mail after sending a consent form for data use and received the signed form. Data from the Korea Health Panel Survey did not include personal or identifiable information. Sample households were selected using 90% of the 2005 Population and Housing Census data as its sampling frame, and the household members of the selected sample households underwent a consent process to be thoroughly investigated. The survey population included 6,408 households and 17,184 members. Data were collected from February 2017 to June 2017 by researchers using computer-assisted personal interviews (CAPI).

In this study, a basic questionnaire survey of households, additional surveys of adult household members, and questions about unmet healthcare needs were answered. In total, 1,151 female baby boomers (born between 1955 and 1963) were included in the final analysis.

### Study tools

Andersen’s behavioral model for health service use is widely used because it considers both individual and environmental characteristics when investigating the determinants of health service use [10].

In this study, independent variables were set as predisposing, enabling, and need factors by applying Andersen’s behavioral model of health service use, as explained below.

#### Predisposing factors

According to Andersen’s behavioral model of health service use, predisposing factors are those associated with unchangeable characteristics that are essentially given to individuals or households. In other words, predisposing factors are characteristics that individuals already have, regardless of their will, before they develop healthcare needs. These include sociodemographic characteristics, such as age and gender, and socioeconomic factors, such as education level [10]. In this study, predisposing factors included age, spouse, and educational level in two age groups (the 50s and 60s). Regarding marital status, being married was classified as ‘Yes,’ whereas bereavement, separation, divorce, and unmarried were classified as ‘none.’ Education level was classified as a) middle school graduates and high school graduates and b) two-year college graduates.

#### Enabling factors

According to Andersen’s behavioral model of health service use, enabling factors are variable factors that affect healthcare needs and utilization; that is, they are factors regarding means and abilities that allow the use of healthcare services, including income level, family resources, and socioeconomic factors [10]. In this study, the enabling factors included health insurance type, economic activity, residential district, and income level. Health insurance types were classified as national health insurance and medical benefits. Economic activity was classified as ‘Yes’ if the participants were currently employed and ‘No’ if they were not. The residential districts were classified into urban and rural areas. Income level was classified using five quintiles based on the income as follows: ‘low’ for the first quintile, ‘moderate’ for the second to fourth quintile, and ‘high’ for the fifth quintile.

#### Need factors

According to Andersen’s behavioral model of health service use, need factors are variables that show healthcare needs, that is, physiological and psychological factors associated with an individual’s disability or disease that directly require the use of healthcare services [10]. In this study, need factors included smoking, drinking, sleep duration, chronic disease, vision problems, hearing problems, eating problems, activity limitations, depression, suicidal ideation, stress, frustration, and anxiety. Smoking was classified as ‘non-smokers’ if individuals had smoked but had quit smoking generally or had never smoked and ‘smokers’ for other cases. Drinking was classified into ‘non-drinkers’ for individuals who did not have a single glass in the past year or who had never had a drink, and ‘drinkers’ for other cases. Regarding sleep duration, the average amount of sleep per day was calculated and used. Based on the recommendation by the American Academy of Sleep Medicine (AASM) of more than seven hours of sleep for adults [23], sleeping was classified as ‘standard’ if individuals slept for 7–8 hours and ‘non-standard’ if the sleep duration is less than or more than the standard time (7–8 hours). Chronic disease was classified as ‘Yes’ if it had lasted for more than three months, otherwise ‘no.’ Regarding vision and hearing problems, ‘hearing or seeing very little’ and ‘hearing or seeing nothing at all’ were classified as ‘Yes,’ otherwise ‘no.’ Eating problems were classified as ‘Yes’ or ‘No.’ If one answered the question, “Have you had a hard time chewing food because of oral and dental problems in the past year?” with either ‘sometimes’ or ‘often,’ the case was classified as ‘Yes,’ otherwise ‘no.’ Activity limitation was classified based on the case when the usual activity, social or leisure life, or family activity was limited because of disease or injury. Depression was classified into ‘Yes’ or ‘No’ for the question, ‘Have you felt sad or unhappy such that it almost disrupted your daily life for at least two consecutive weeks over the last year? Suicidal ideation was classified into ‘Yes’ or ‘No’ for the question ‘Have you had thoughts of dying in the last year? Stress, frustration, and anxiety were measured using the following: five points were given for those who experienced stress, frustration, or anxiety all the time in the last month, four points for almost all the time, three points for several times, two points for sometimes, and one point for never.

Regarding the dependent variables, unmet healthcare needs were considered present if the respondent answered ‘yes’ to the following: “I did not receive medical care at least even once, although I needed to go to a hospital or clinic for medical treatment or examination in the last year.”

### Data analysis

All data were analyzed using the SPSS WIN 27 program. Data were analyzed using t-tests, analysis of variance (ANOVA), Pearson’s correlation coefficient, and multivariate regression. The current status and cases of participants’ unmet healthcare needs were expressed as frequencies and percentages. Frequency, percentage, chi-square test, and t-test were used to characterize unmet healthcare needs because of the participants’ predisposing, enabling, and need factors. The characteristics of predisposing, enabling, and need factors that affect participants’ unmet healthcare needs were investigated using univariate logistic regression analysis.

Variables with a significance level of ≤0.05 in the univariate logistic regression analysis were used to conduct multiple logistic regression analysis. Subsequently, variables with a significance level of ≤0.05 in the chi-square tests and t-tests were used to conduct multiple logistic regression analysis. The results were presented as odds ratios (OR) and 95% confidence intervals (CI).

## Results

### Current status and causes of unmet healthcare needs

The current status and causes of unmet healthcare needs are shown in Table 1. A total of 1,151 participants were included in the study, of which 128 (11.1%) answered that they experienced unmet healthcare needs.

**Table 1:** Unmet healthcare needs of subjects (n = 1,151).

Variable	Category	n	%
Unmet healthcare needs	No	**1,023**	**88.9**
	Yes	**128**	**11.1**
Reason for unmet healthcare needs	No time to visit	**51**	**39.8**
	Minor symptoms	**41**	**32.0**
	Economic reasons (Burden of treatment costs)	**26**	**20.3**
	Inconvenient behavior, Health reasons	**5**	**3.9**
	Did not know where to go (Lack of information)	**2**	**1.6**
	Hospitals are too far away	**1**	**0.8**
	I don’t have anyone to look after her	**1**	**0.8**
	Others	**1**	**0.8**

Regarding the causes of unmet healthcare needs, ‘no time to visit’ was the highest cause, accounting for 39.8% of all causes, followed by ‘mild symptoms’ and ‘economic reasons (high cost of medical treatment),’ accounting for 32.0% and 20.3%, respectively.’

### Characteristics of unmet healthcare needs

Table 2 shows the differences in predisposing, enabling, and need factors of unmet and meeting healthcare needs. Among the predisposing factors, participants (17.6%) without a spouse experienced more unmet healthcare needs, but there were no differences in age and education level. In the enabling factors, the participants (16.8%) with a ‘low’ income level experienced more unmet healthcare needs, but there were no differences in health insurance type, economic activity, and residential district. Among the need factors, smokers (32.0%), participants with vision (16.5%), eating (16.5%), activity limitation (27.9%), and suicidal ideation (26.3%) experienced more unmet healthcare needs. Participants with a high level of stress (2.16 points), frustration (1.61 points), or anxiety (2.02 points) experienced more unmet healthcare needs. There were no differences in drinking, sleep duration, chronic diseases, hearing problems, and depression.

**Table 2:** Unmet healthcare needs with demographic characteristics (n = 1,151).

Characteristics		Categories	Total	Met needs	Unmet needs	χ^2^	*p*
			n (%)	n(%)/M±SD	n(%)/M±SD		
Predisposing factors	Age	50~59	747 (64.9)	665 (89.0)	82 (11.0)	0.04	(.833)
		60~64	404 (35.1)	358 (88.6)	46 (11.4)		
	Spouse	Yes	964 (83.8)	869 (90.1)	95 (9.9)	9.62	(.002)
		No	187 (16.2)	154 (82.4)	33 (17.6)		
	Education	≤Middle school	506 (44.0)	444 (87.7)	62 (12.3)	3.52	(.172)
		High school	475 (41.3)	421 (88.6)	54 (11.4)		
		≥College	170 (14.8)	158 (92.9)	12 (7.1)		
Enabling factors	Insurance type	Medical Insurance	1,128 (98.0)	1,004 (89.0)	124 (11.0)	0.93	(.334)
		Medicaid	23 (2.0)	19 (82.6)	4 (17.4)		
	Economic activity	Yes	721 (62.6)	632 (87.7)	89 (12.3)	2.92	(.087)
		No	430 (37.4)	391 (90.9)	39 (9.1)		
	Region	Urban	517 (44.9)	461 (89.2)	56 (10.8)	0.08	(.778)
		Rural	634 (55.1)	562 (88.6)	72 (11.4)		
	Total household income	High	306 (26.6)	284 (92.8)	22 (7.2)	8.52	(.014)
		Moderate	750 (65.2)	660 (88.0)	90 (12.0)		
		Low	95 (8.3)	79 (83.2)	16 (16.8)		
Need factors	Smoking	No	1,126 (97.8)	1,006 (89.3)	120 (10.7)	11.27	(.001)
		Yes	25 (2.2)	17 (68.0)	8 (32.0)		
	Drinking	No	537 (46.7)	473 (88.1)	64 (11.9)	0.65	(.421)
		Yes	614 (53.3)	550 (89.6)	64 (10.4)		
	Sleeping time	Standard	471 (40.9)	423 (89.8)	48 (10.2)	0.70	(.404)
		Non-standard	680 (59.1)	600 (88.2)	80 (11.8)		
	Chronic disease	No	972 (84.4)	859 (88.4)	113 (11.6)	1.61	(.204)
		Yes	179 (15.6)	164 (91.6)	15 (8.4)		
	Vision problem	No	770 (66.9)	705 (91.6)	65 (8.4)	16.89	(<.001)
		Yes	381 (33.1)	318 (83.5)	63 (16.5)		
	Hearing problem	No	1,079 (93.7)	964 (89.3)	115 (10.7)	3.74	(.053)
		Yes	72 (6.3)	59 (81.9)	13 (18.1)		
	Eating problem	No	617 (53.6)	577 (93.5)	40 (6.5)	28.94	(<.001)
		Yes	534 (46.4)	446 (83.5)	88 (16.5)		
	Activity restriction	No	1,108 (96.3)	992 (89.5)	116 (10.5)	12.73	(<.001)
		Yes	43 (3.7)	31 (72.1)	12 (27.9)		
	Depression	No	1,067 (92.7)	952 (89.2)	115 (10.8)	1.74	(.187)
		Yes	84 (7.3)	71 (84.5)	13 (15.5)		
	Suicidal ideation	No	1,113 (96.7)	995 (89.4)	118 (10.6)	9.18	(.002)
		Yes	38 (3.3)	28 (73.7)	10 (26.3)		
	Stress		1.71±0.91	1.65±0.87	2.16±1.04	-6.00	(<.001)
	Frustration		1.30±0.62	1.26±0.59	1.61±0.77	-4.92	(<.001)
	Anxiety		1.63±0.84	1.58±0.81	2.02±1.00	-5.59	(<.001)

*Note*. M, Mean; SD, Standard Deviation

### Factors affecting unmet healthcare needs

To investigate factors affecting unmet healthcare needs in female baby boomers, multivariate logistic regression analysis was performed by using variables with a significance level of ≤0.05 in univariate logistic regression analysis. Multicollinearity between the independent variables was confirmed prior to the analysis. Since the variance inflation factor values were not greater than 2.0 (1.08–1.73), tolerance limits were all ≥0.1 (0.58–0.94), and the condition index was not greater than 15 (1.00–8.69); multicollinearity was not suspected. Multivariate logistic regression analysis of factors affecting unmet healthcare needs in female baby boomers is presented in Table 3. Univariate logistic regression analysis showed that the p-values for spouses in the predisposing factors, income level in the enabling factors, smoking, vision and eating problems, activity limitation, suicidal ideation, stress, frustration, and anxiety were all at a significance level of ≤0.05.

**Table 3:** Factors associated with unmet healthcare needs (n = 1,151).

Variables (reference group)			OR	(95% CI)	Wald	*p*
Predisposing factors	Spouse (ref: Yes)	No	1.63	(1.01–2.66)	3.93	.047
Enabling factors	Total household income (ref: High)	Moderate	1.37	(0.82–2.27)	1.47	.226
		Low	1.15	(0.52–2.56)	0.13	.723
Need factors	Smoking (ref: No)	Yes	2.31	(0.87–6.14)	2.82	.093
	Vision problem (ref: No)	Yes	1.45	(0.97–2.18)	3.20	.073
	Eating problem (ref: No)	Yes	2.33	(1.52–3.55)	15.22	<.001
	Activity restriction (ref: No)	Yes	1.66	(0.74–3.72)	1.52	.218
	Suicidal ideation (ref: No)	Yes	1.06	(0.45–2.50)	0.02	.896
	Stress		1.31	(1.03–1.67)	4.94	.026
	Frustration		1.21	(0.86–1.70)	1.16	.282
	Anxiety		1.22	(0.95–1.56)	2.43	.119

Log-likelihood = 720.97, χ² (*p*) = 81,79 (<.001)

Negelkerke R^2^ =.14, Hosmer-Lemeshow’s Goodness of fit *p* = .217

*Note*. OR, Odds Ratio; CI, Confidence Interval

Spouses, eating disorders, and stress were identified as factors affecting unmet healthcare needs by multivariate logistic regression analysis using these variables. The Hosmer-Lemeshow model fit test revealed that the regression analysis model was appropriate (χ^2^ = 10.74, p = .217). It was found that 14.0% of the dependent variable could be explained by the regression model (Nagelkerke R^2^ = .140).

Participants without a spouse experienced 1.63-fold unmet healthcare needs (CI = 1.01–2.66, p = .047) more than those with a spouse did. Participants with eating problems experienced 2.33-fold unmet healthcare needs (CI = 1.52–3.55, p < .001) more than those without eating problems. Participants with stress experienced 1.31-fold unmet healthcare needs (CI = 1.03–1.67, p = .026) more than those without stress.

## Discussion

This study identified factors affecting unmet healthcare needs in female baby boomers using data from the Korea Health Panel Survey 2017 based on Andersen’s behavioral model of health service use. The findings showed that 11.1% of female baby boomers (aged between 59 and 67 years) experienced unmet healthcare needs. Since no previous study has been conducted on female baby boomers to date, it cannot be compared directly. The comparative group included older adults aged ≥65 years. A study by Lee and Hug reported that 17.9% of older female adults aged ≥65 years experienced unmet healthcare needs [24], which was higher than the proportion in this study. This aligns with the results that the higher the age, the higher the unmet healthcare needs in older female adults [25]. Further studies should be conducted to determine how healthcare service utilization differs between female baby boomers who enter the early phase of old age and previous older adults and investigate factors affecting unmet healthcare needs. Based on these findings, a policy on improving access to healthcare services should be established.

The current study showed that factors affecting unmet healthcare needs in female baby boomers were the ‘predisposing factors’ including sociodemographic and socioeconomic characteristics of individuals, and the ‘need factors’ showing medical needs by subjective recognition or professional determination of health condition. Each factor is further discussed below.

First, among the predisposing factors, women without a spouse showed 1.6-fold higher unmet healthcare needs than women with a spouse. A spouse is a human source who can monitor each other’s health condition at all times and can accompany one with access to a medical institution. However, the absence of a spouse limits access to medical institutions when healthcare services are required. This is consistent with a previous study that reported that the absence of a spouse was a factor affecting unmet healthcare needs [26], aligning with this study’s findings. Moreover, the absence of a spouse is associated with difficulty in accessibility to healthcare services and economic difficulties in female baby boomers. Female baby boomers experience job insecurity and are more economically vulnerable than male baby boomers [26, 27]. Notably, 20.3% of the female baby boomers in this study answered that the cause of their unmet healthcare needs was economic difficulties. Therefore, a community support system should be developed, and an employment program should be implemented so that female baby boomers can access healthcare services. Female baby boomers have higher education levels than older adults and are more interested in health [28]. A system should be developed for them to consult regarding health problems. Moreover, the government should efficiently operate the public health system so that it can be involved in self-health management education programs within the community to promote health.

Second, unmet healthcare needs among female baby boomers are affected by eating problems. Female baby boomers with eating problems had 2.33-fold higher unmet healthcare needs than those without these problems. Eating problems may not be a direct cause for unmet healthcare needs. However, eating in older adults is an indicator that can measure current physical and mental health conditions and can be associated with emotional states, such as depression and anxiety [29]. Furthermore, eating is essential to maintaining and promoting future health and critical in maintaining older adults’ quality of life. A subsequent study should investigate how eating problems in female baby boomers affect unmet healthcare needs and suggest eating and nutrition education to maintain their essential health.

The last factor affecting unmet healthcare needs in female baby boomers for this study was stress among the need factors. Female baby boomers with stress showed a 1.31-fold increase in unmet healthcare needs. This aligns with a previous study’s results that perceived stress affects unmet healthcare needs [27, 30, 31]. When female baby boomers transition from middle age to the early phase of old age, they can experience a sense of crisis because of their surroundings. To date, social discussions have primarily focused on the work, retirement, income, and caregiver role of male baby boomers, while little has been examined about the life of female baby boomers [20]. Female baby boomers face physical deterioration [32] and a higher proportion of chronic disease or depression than those reported in male baby boomers [33]. Moreover, they have not been prepared for their old age in every domain, such as economy, health, leisure life, and social participation, because of economic difficulty, lack of time, and insufficient information compared to male baby boomers [20, 33]. Accordingly, female baby boomers are exposed to various stressful situations and experience helplessness and frustration, affecting their unmet healthcare needs. Further studies should be conducted to identify emotional problems in depth and investigate the causality between mental health problems and unmet healthcare needs among female baby boomers.

This study investigated the factors affecting unmet healthcare needs in female baby boomers using the Korean Health Panel Survey. Moreover, the study is significant because measures were sought to reduce the difference between healthcare needs and healthcare utilization. Nevertheless, this study has some limitations. First, since participants had evaluated unmet healthcare needs using a self-reported questionnaire, subjective judgment was involved. Second, the unmet healthcare needs measurement index included ‘Yes’ or ‘No’ questions. Thus, there is a limit to the complete reflection of the level of healthcare utilization. Third, this is a cross-sectional study. Since it was difficult to determine the temporal order relationship between unmet healthcare needs and influencing factors in female baby boomers, causality could not be identified using the data in this study.

Further studies should be conducted to periodically analyze unmet healthcare needs trends and influencing factors in female baby boomers and to develop a plan to improve their access to healthcare services.

## Conclusion

This study investigated factors affecting unmet healthcare needs in female baby boomers using Andersen’s behavioral model of health service use. Female baby boomers experienced more unmet healthcare needs when they had no spouse (1.63 times), eating problems (2.33 times), and stress (1.31 times). Social policies related to unmet healthcare needs have been advanced and centered on older adults until now. Hence, further studies should be conducted to confirm the factors affecting unmet healthcare needs by periodically investigating the healthcare needs of baby boomers. Furthermore, a strategy to improve access to healthcare services by creating jobs and social support should be developed for those who retire without preparing to enter old age.

## Supporting information

S1 Checklist STROBE statement—Checklist of items that should be included in reports of *cross-sectional studies*. (DOCX)
